# Hypoxia‐Induced Suppression of FAM99A and FAM99B Contributes to the Development and Glucose Metabolic Reprogramming of Hepatocellular Carcinoma

**DOI:** 10.1096/fj.202501058R

**Published:** 2025-07-22

**Authors:** Cang‐sang Song, Guo‐hui Wang, Pan‐pan Mao, Han‐shu Zhang, Lu Liu, Xue‐jiao Ma, Xing‐de Li, Yang Zhang

**Affiliations:** ^1^ Department of Pharmacy The First People's Hospital of Kunming City & Calmette Affiliated Hospital of Kunming Medical University Kunming Yunnan China; ^2^ Department of Pharmacy The Third People's Hospital of Yunnan Province Kunming Yunnan China

**Keywords:** bioinformatics, competing endogenous RNA, glucose metabolic reprogramming, hepatocellular carcinoma, hypoxia, LncRNA

## Abstract

Hepatocellular carcinoma (HCC) is a highly aggressive and highly malignant cancer. Glucose metabolic reprogramming provides sufficient ATP to support HCC's rapid proliferation and invasion. Consequently, this study intends to investigate the effects of FAM99A and FAM99B on glucose metabolic reprogramming, and provide new insights for HCC treatment. Changes in malignant phenotypes and glycolysis‐related indices of HCC cells (HCCLM3 and HEPG2) were assessed after exogenous regulation of FAM99A and FAM99B under hypoxic conditions. Oxygen consumption rate (OCR), extracellular acidification rate (ECAR), and glycolytic proton efflux rate (glycoPER) were measured using the Seahorse XF Glycolysis Rate Assay Kit (103344‐100, Agilent). HCCLM3 cells were subjected to transcriptome and smallRNA sequencing to identify differentially expressed genes (DEGs) and miRNAs (DE‐miRNAs) associated with FAM99A and FAM99B. Under hypoxic conditions, the expression of FAM99A and FAM99B was significantly downregulated in HCC cells. Overexpression of FAM99A or FAM99B significantly inhibited HCC cell proliferation, wound healing, and invasion. Moreover, they effectively decreased intracellular glucose, extracellular lactate, ATP, glycolysis‐related enzymes, ECAR, and glycoPER, and increased pH, extracellular glucose, and mitoOCR/glycoPER. A total of 31 DEGs and 15 DE‐miRNAs were present in HCCLM3 cells overexpressing FAM99A, and 375 DEGs and 68 DE‐miRNAs were identified in HCCLM3 cells overexpressing FAM99B. These DEGs and DE‐miRNA targets were involved in cell cycle, apoptosis, metastasis, extracellular matrix remodeling, and metabolic reprogramming. The FAM99B‐associated ceRNA network contained one DE‐miRNA and 10 DEGs, and their expression differences were consistent with the sequencing results. Hypoxia‐induced suppression of FAM99A and FAM99B facilitates proliferation, metastasis, and glucose metabolic reprogramming of HCC.

## Graphical abstract

FAM99A and FAM99B suppress glycolysis, proliferation, invasion, and migration of hepatocellular carcinoma under hypoxic conditions, which may be related to the miR‐1291‐mediated ceRNA mechanism.

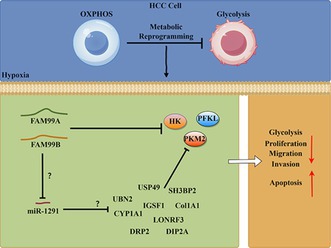

**Abbreviations**
DEGs: differentially expressed genes
DE‐miRNAs: differentially expressed miRNAs
FC: fold change
HCC: hepatocellular carcinoma
KD: knockdown
L‐LA: l‐lactic acid
OV: overexpression
PPI: protein–protein interaction
ShRNA: short hairpin RNA

## 1 Introduction

Hepatocellular carcinoma (HCC) is the most common malignant tumor worldwide, accounting for more than 80% of all liver cancers. It is mainly induced by chronic liver diseases such as cirrhosis, chronic hepatitis, hepatitis B virus and hepatitis C virus infection, alcohol abuse, and genetics [1]. According to statistics, there are about 780 000 new cases of HCC each year worldwide, of which China accounts for nearly half [2]. Moreover, HCC has a high mortality rate, being the third leading cause of cancer death worldwide, and the 5‐year relative survival rate is only about 21% [3]. This is mainly because the early symptoms of HCC are not obvious, and most patients are already at an advanced stage when diagnosed [4]. Surgical resection, liver transplantation, radiotherapy, and/or chemotherapy are the main treatments for HCC; nevertheless, patient prognosis is not satisfactory [4, 5, 6]. Hence, there is an urgent need to further understand the mechanisms of HCC progression in order to develop new therapeutic strategies.

Glucose metabolism is an essential process for cell survival, providing energy to maintain glucose homeostasis and various physiological activities in the body [7]. Tumor cells are significantly different from normal cells in terms of glucose metabolism. Normal cells produce adenosine triphosphate (ATP) mainly through oxidative phosphorylation of mitochondria to meet cell survival and functional needs [7]. Tumor cells tend to produce ATP efficiently through the glycolytic metabolic pathway and produce large amounts of organic acids such as lactic acid and pyruvic acid [8, 9]. This metabolic approach not only provides tumor cells with sufficient energy and raw materials for growth, but also reduces their dependence on oxygen, allowing them to continue to grow in a hypoxic environment [8, 10]. Glucose metabolic reprogramming is an adaptive response of tumor cells to environmental factors and nutrient uptake, which makes it a potential target for tumor therapy [11, 12]. LncRNA FAM99A and LncRNA FAM99B (hereafter FAM99A and FAM99B) are encoded by two adjacent genes located on human chromosome 11, respectively, and are significantly more expressed in human liver and placenta than in other tissues [13]. In pre‐eclampsia, FAM99A was proven to be significantly downregulated and to regulate apoptosis, migration, and invasion of trophoblast cells [14, 15]. Previous studies have demonstrated that FAM99A and FAM99B, promising prognostic biomarkers, are significantly under‐expressed in HCC and significantly inhibit HCC cell proliferation, migration, and invasion [16, 17]. It is worth mentioning that FAM99A has been shown to be associated with hepatic and placental metabolism [18, 19]. Zhao et al. [20] indicated that hypoxic conditions can regulate FAM99A expression and that downregulation of FAM99A expression facilitates the HCC cell metastasis. It is suggested that FAM99A may be associated with the metabolic reprogramming of HCC in a hypoxic environment. Furthermore, the mechanism of the role of FAM99B, a lncRNA extremely similar to FAM99A, in the metabolic reprogramming of HCC remains to be investigated.

In summary, the present study was designed to investigate the effects of FAM99A and FAM99B on the glucose metabolic reprogramming of HCC in vitro and to reveal the potential mechanisms of FAM99A and FAM99B by transcriptome and smallRNA sequencing. The aim is to provide new drug targets and individualized treatment strategies for the treatment and diagnosis of HCC.

## 2 Methods and Materials

### 2.1 Cell Culture and Hypoxia Treatment

HCC cells (HEPG2, SK‐1, and HCCLM3) were obtained from Wuhan Pricella Lifescience Co. Ltd. HEPG2, SK‐1, and HCCLM3 cells were cultured in medium consisting of 88% Dulbecco's modified Eagle medium (DMEM) (G4515; Servicebio, China), 10% FBS (10099‐141; Gibco, USA), 1% double antibodies, and 1% glutamine, and cultured in a CL191C incubator (Crystal Technology, USA) at 37°C. For the hypoxia treatment, cells were incubated in an environment consisting of 2% O_2_, 93% N_2_, and 5% CO_2_ at about 70% cell fusion. Cells were collected after 48 h for subsequent assays.

### 2.2 Cell Transfection

The short hairpin RNA (shRNA) lentivirus against FAM99A and FAM99B and its corresponding negative control were designed and packaged by Genomeditech (Shanghai) Co. Ltd. The vector for the shRNA was GM‐19167 RNAi. Similarly, the lentiviral‐encapsulated PGMLV‐CMV‐MCS‐EF1‐ZsGreen1‐T2A‐Puro vector overexpressing FAM99A and FAM99B and its negative control were designed and synthesized by Genomeditech. The target sequences of shRNA and lentiviral titers are shown in Table S1. HCCLM3 cells were randomly divided into OV‐NC, OV‐FAM99A, and OV‐FAM99B groups. HEPG2 cells were randomly divided into KD‐NC, KD‐FAM99A, and KD‐FAM99B groups. According to the grouping information, the infection of FAM99A and FAM99B overexpressing lentivirus and FAM99A and FAM99B interfering lentivirus were performed, respectively. After 72 h of infection, the cell medium was changed to medium containing 5 μg/mL puromycin for resistance screening to obtain stable lines. Stable lines were subjected to hypoxia treatment and used for subsequent experiments.

### 2.3 Cell Proliferation Assay

Cell proliferation assays were performed by cell counting kit‐8 (CCK‐8) (CK04; Dojindo Laboratories, Japan) and flow cytometry for detection of cell viability and cycle, respectively. For CCK‐8 assays, HCCLM3 and HEPG2 cells were inoculated in 96‐well plates and incubated at 0, 12 and 24, 48 h, respectively, after which 10 μL of CCK‐8 solution was added to each well. After incubation for 4 h under darkness, the optical density values of each well were detected at 450 nm using an EL800 microplate reader (BioTek, USA). For cell cycle assays, HCCLM3 and HEPG2 cells were digested with 0.25% trypsin and cell precipitates were obtained by centrifugation at 1000 rpm for 5 min. After washing with PBS, cells were added with 0.5 mL of propidium Iodide staining solution. After incubation for 30 min at 37°C without light, the proportion of cells in G1, S and G2 periods was detected at an excitation wavelength of 488 nm using a Novocyte advanteon flow cytometer (Agilent, USA).

### 2.4 Cell Migration and Invasion Assay

The detection of cell migration and invasion was performed by wound healing assay and Transwell assay, respectively. For wound healing experiments, HCCLM3 and HEPG2 cells were digested with 0.25% trypsin, and the cell concentration was adjusted to 2.5 × 10^4^ cells/mL. Cells were inoculated in ibidi scratch inserts (Ibidi, Germany) with 100 μL in each of the left and right wells. The following day, the ibidi scratch insert was removed, DMEM medium was added, and picture ingestion was performed at 0 and 48 h. For Transwell assays, 50 μL Matrigel (346234; Corning, USA) was loaded in the Transwell upper chamber and dried at 37°C for 30 min. Transwell was inoculated with 100 μL of serum‐free medium containing 1 × 10^5^ cells/mL in the upper chamber and 500 μL of DMEM medium containing FBS in the lower chamber. After 48 h of incubation, cells were fixed with 4% polymethylglyoxal for 30 min and stained with crystal violet staining solution for 15 min. Images of wound healing and Transwell assay were collected by BX53 microscopy (Olympus, Japan).

### 2.5 Glucose, Lactic Acid, and ATP Assays

Briefly, the cells were crushed using a VCX 130 ultrasonic cell crusher (Sonics, USA) and the supernatant was prepared. Referring to the instructions of the Glucose kit (A154‐1‐1; Njjcbio, China), l‐lactic acid (L‐LA) content test kit (bc2235; Solarbio), and ATP content test kit (bc0305; Solarbio), the optical density values were measured at 505, 570, and 340 nm on the EL800 microplate reader to determine the cellular contents of glucose, lactic acid, and ATP.

### 2.6 Seahorse Assay

Oxygen consumption rate (OCR), extracellular acidification rate (ECAR), and glycolytic proton efflux rate (glycoPER) were measured in HCCLM3 and HEPG2 cells using the Seahorse XF Glycolysis Rate Assay Kit (103344‐100, Agilent) and Seahorse XFe24 (Agilent). Briefly, HCCLM3 and HEPG2 cells were inoculated into XF24 cell culture plates (3 × 10^4^ cells/well) and cultured for 24 h, followed by hypoxic treatment for 48 h. Prior to measurement, one sensor probe plate was hydrated with Seahorse XF calibration solution. Referring to the kit instructions, Rot/AA (0.5 μmol/L) and 2‐DG (50 mmol/L) were added sequentially to well A with a final volume of 56 μL and well B with a final volume of 62 μL. After calibration, cells were detected and analyzed for OCR, ECAR, and glycoPER using Seahorse XF Glycolytic Rate Assay mode with Seahorse XFe24 and Wave Desktop software.

### 2.7 Western Blotting Assay

Briefly, HCCLM3 and HEPG2 cells were subjected to total protein extraction and total protein concentration determination using RIPA lysate (P0013C; Beyotime, China) and bicinchoninic acid (BCA) protein concentration determination kit (P0012; Beyotime), respectively. Samples were electrophoresed in 10% SDS‐PAGE membranes and transferred to PVDF membranes. PVDF membranes were blocked with 5% BSA and incubated with Anti‐Hexokinase (HK) (ab150423; 1:1000; Abcam, UK), Anti‐Phosphofructokinase (PFKL) (ab97443; 1:1000; Abcam), Anti‐Pyruvate kinase M2 (PKM) (ab150377; 1:10 000; Abcam) and Anti‐GAPDH (P30008M; 1:1000; Abmart, China) antibodies at 4°C. The following day, polyvinylidene fluoride (PVDF) membranes were treated with anti‐rabbit IgG, HRP‐linked Antibody (7074; 1:2000; CST, USA). After color development by electrochemiluminescence (ECL) Chemiluminescence Solution (P1020‐1; ApplyGen, China), images were collected using a 5200 Multi gel imaging system (Tanon, China) and grayscale values of gel blots were determined by Image J software [21].

### 2.8 RT‐qPCR Assay

The extraction of total RNA and determination of total RNA concentration in HCCLM3 and HEPG2 cells were performed by Trizol Reagent (15596026; Lifetech, USA) and EzDrop 1000 UV spectrophotometer (Blue Ray, China), respectively. The reverse transcription of total RNA and amplification of miR‐1291 were performed in 7500 Real Time PCR System (ABI, USA) with reference to the instructions of miDETECT A Track miRNA qRT‐PCR Starter Kit (C10712‐1; RiboBio, China). LncRNA and mRNA amplification was performed by the riboSCRIPT mRNA/lncRNA qRT‐PCR Starter Kit (C11030‐2; RiboBio). The 2(−Delta Delta C(T)) method [22] was used to calculate the relative expression levels of each target gene. The primer sequences are shown in Table S2.

### 2.9 Transcriptome and SmallRNA Sequencing

Transcriptome and SmallRNA Sequencing of HCCLM3 cells in OV‐NC, OV‐FAM99A and OV‐FAM99B groups was done by oebiotech (Shanghai) Co. Ltd. A total of 61.45 G of valid data were obtained from transcriptome sequencing, and the distribution of valid data for each sample ranged from 6.66 to 6.94 G. The distribution of Q30 bases ranged from 91.38% to 91.66%, and the matching rate was 94.11% to 94.67%. A total of 208.72 M of valid data volume was obtained by SmallRNA Sequencing, and the valid data volume of each sample was distributed from 20.0 to 24.71 M. The total number of known miRNAs on all samples matched was 1718, and the matching rate was distributed from 35.01% to 42.15%. The acquisition of differentially expressed genes (DEGs) and differentially expressed miRNAs (DE‐miRNAs) were performed based on the DESeq2 R package [23]. The threshold is *p* < 0.05 after calibration and log_2_ Fold Change (FC) > 1 for absolute values. The target genes of DE‐miRNAs were predicted from the miRNAda database [24]. The enrichment analysis of DEGs and DE‐miRNA targets was performed by hypergeometric algorithms based on Gene Ontology (GO) [25] and Kyoto Encyclopedia of Genes and Genomes (KEGG) [26] databases. The protein–protein interaction (PPI) network of DEGs was constructed using the String database [27] and CytoScape software [28].

### 2.10 Statistical Analysis

Data were statistically analyzed by GraphPad Prism software (GraphPad, USA). Briefly, Student's *t* and Mann–Whitney *U* tests were used for comparison between two groups, and one‐way ANOVA and Kruskal–Wallis *H* tests were used for comparison between multiple groups. *p* < 0.05 was considered statistically significant.

## 3 Result

### 3.1 FAM99A and FAM99B Inhibit Proliferation, Migration, and Invasion of Hepatocellular Carcinoma Cells Under Hypoxic Conditions

**FIGURE 1:**
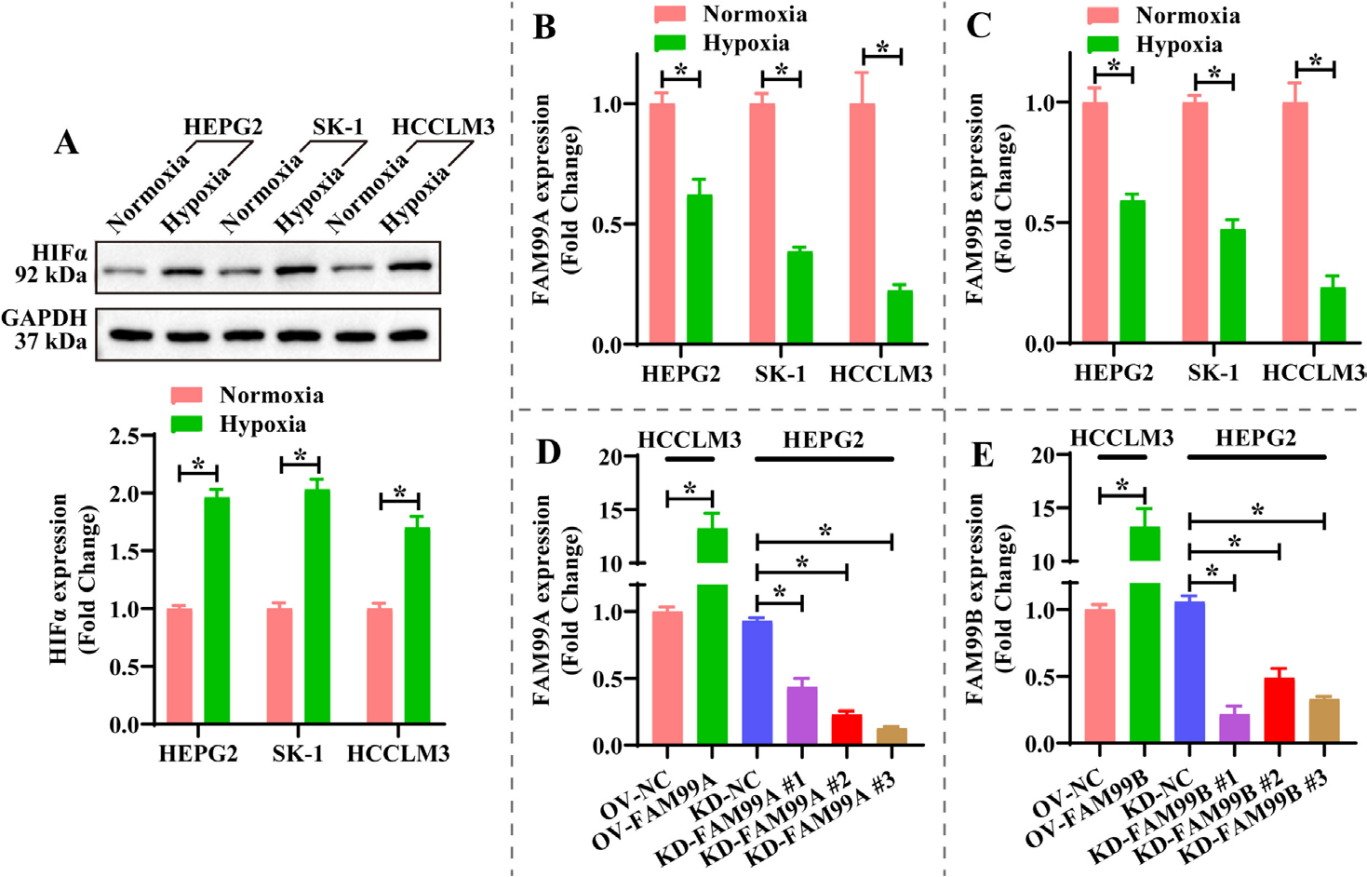
Hypoxia inhibits FAM99A and FAM99B expression in hepatocellular carcinoma. (A) Representative pictures of HIFα gel blot in hepatocellular carcinoma cells (HEPG2, SK‐1, and HCCLM3) and statistical analysis of grayscale values. Original gel blot as shown in the Supporting Information. (B, C) Expression of FAM99A and FAM99B under normoxic and hypoxic conditions was measured by RT‐qPCR. (D, E) Expression of (D) FAM99A and (E) FAM99B in HEPG2 and HCCLM3 cells after exogenous modulation was detected by RT‐qPCR. Data that conformed to normal distribution were statistically analyzed using Student's *t* test or one‐way ANOVA; otherwise, the Mann–Whitney *U* test or Kruskal–Wallis *H* test was used. *N* = 3, and * represents *p* < 0.05.

**FIGURE 2:**
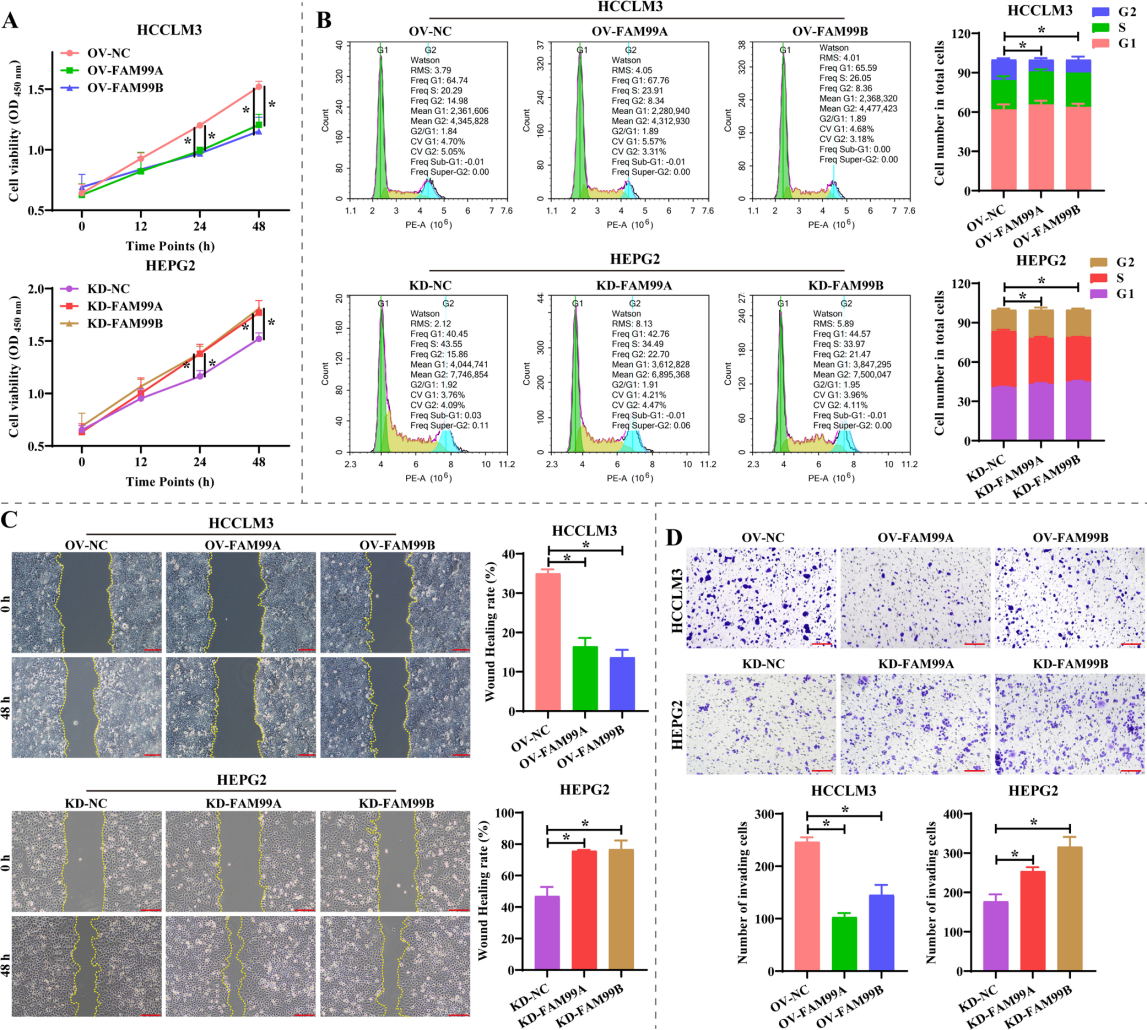
FAM99A and FAM99B inhibit proliferation, migration, and invasion of hepatocellular carcinoma cells under hypoxic conditions. (A) The viability of HEPG2 and HCCLM3 cells at different time points (0, 12, 24, and 48 h) was detected based on the CCK‐8 assay. (B) The proportion of HEPG2 and HCCLM3 cells in G1, S, and G2 phases was detected by flow cytometry. (C) Representative images of wound healing in HEPG2 and HCCLM3 cells at 0 and 48 h, and statistical analysis of the proportion of wound healing. Scale bar: 200 μm. (D) The effect of FAM99A and FAM99B on HEPG2 and HCCLM3 cell invasion was examined by the Tranwell assay. Scale bar: 200 μm. Data that conformed to normal distribution were statistically analyzed using one‐way ANOVA; otherwise, the Kruskal–Wallis *H* test was used. *N* = 3, and * represents *p* < 0.05.

It is well known that hypoxia‐mediated glucose metabolic reprogramming is an important factor in tumor progression; therefore, we examined the effect of hypoxia on FAM99A and FAM99B expression in HCC cells (HEPG2, SK‐1 and HCCLM3). Western blotting revealed that the expression of HIFα was significantly increased in HEPG2, SK‐1, and HCCLM3 cells under hypoxic conditions (Figure 1A, *p* < 0.05), which indicated that the hypoxic environment of HCC cells was successfully constructed. Notably, the expression of both FAM99A and FAM99B was significantly downregulated in HEPG2, SK‐1, and HCCLM3 cells in a hypoxic environment and was most significant in HCCLM3 (Figure 1B,C, *p* < 0.05). It is suggested that FAM99A and FAM99B may be associated with hypoxia‐mediated malignant biological behavior. Thus, we exogenously regulated the expression of FAM99A and FAM99B in HCCLM3 and HEPG2 to explore their effects on the malignant phenotype of HCC cells. As displayed in Figure 1D,E, different lentiviruses were effective in increasing or decreasing the expression of FAM99A and FAM99B in HCCLM3 and HEPG2 under hypoxic conditions, and the knockdown effect of FAM99A #3 and FAM99B #1 was the most significant (*p* < 0.05), so they were selected for subsequent experiments. Overexpression of FAM99A and FAM99B in hypoxic HCCLM3 cells significantly attenuated cell viability and G2 phase cell ratio, while knockdown of FAM99A and FAM99B in HEPG2 cells yielded the opposite results (Figure 2A,B, *p* < 0.05). It is notable that overexpression of FAM99A and FAM99B significantly inhibited the wound healing ratio and the number of invading cells in hypoxic HCCLM3 cells, and knockdown of FAM99A and FAM99B facilitated wound healing and invasion in HEPG2 cells (Figure 2C,D, *p* < 0.05). The above results indicated that the expression of FAM99A and FAM99B was suppressed under hypoxic conditions, which benefited the proliferation, migration, and invasion of HCC cells.

### 3.2 FAM99A and FAM99B Attenuate the Glycolysis Rate in Hepatocellular Carcinoma Cells Under Hypoxic Conditions

**FIGURE 3:**
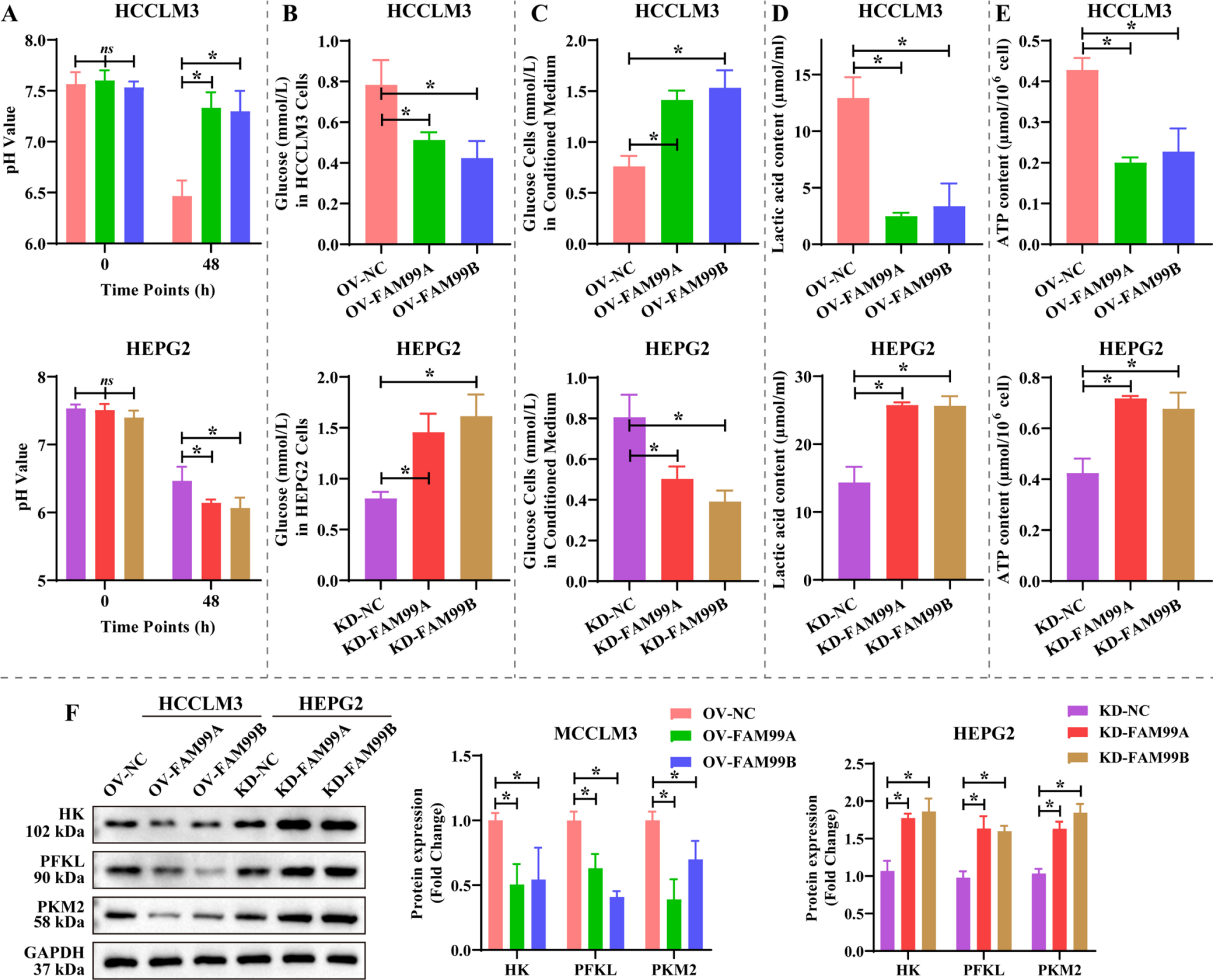
FAM99A and FAM99B alleviate glucose metabolic reprogramming in hepatocellular carcinoma cells under hypoxic conditions. (A) pH values of HEPG2 and HCCLM3 cell supernatants at 0 and 48 h. (B, C) Levels of glucose in (B) cells (HCCLM3 and HEPG2) and (C) corresponding conditioned medium. (D, E) Levels of (D) lactic acid and (E) ATP in HEPG2 and HCCLM3 cells after 48 h of transfection. (F) The effect of FAM99A and FAM99B on the expression of glycolysis‐related proteins (HK, PFKL, and PKM2) under hypoxic conditions was examined by western blotting. Data that conformed to normal distribution were statistically analyzed using one‐way ANOVA; otherwise, the Kruskal–Wallis *H* test was used. *N* = 3, and * represents *p* < 0.05. Original gel blot as shown in the Supporting Information.

**FIGURE 4:**
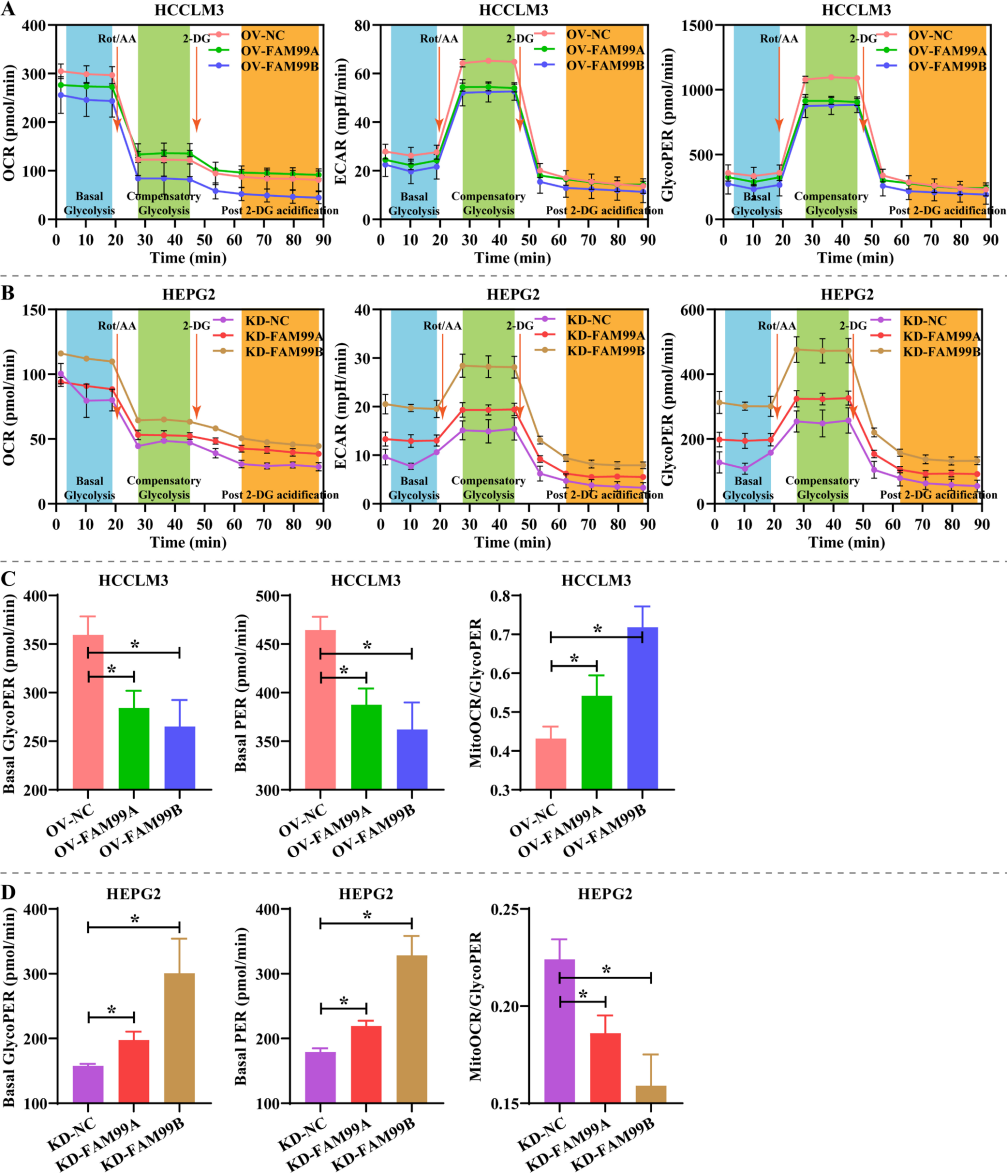
FAM99A and FAM99B attenuate the glycolysis rate in hepatocellular carcinoma cells under hypoxic conditions. (A, B) Seahorse assay was conducted to assay the effects of FAM99A and FAM99B on oxygen consumption rate (OCR), extracellular acidification rate (ECAR), and glycolytic proton efflux rate (glycoPER) in (A) HCCLM3 and (B) HEPG2 cells. (C, D) Differential analysis of basal glycoPER, basal PER, and mitoOCR/glycoPER in (C) HCCLM3 and (D) HEPG2 cells. Data that conformed to normal distribution were statistically analyzed using one‐way ANOVA; otherwise, Kruskal–Wallis *H* test was used. *N* = 3, and * represents *p* < 0.05.

Because the expression of FAM99A and FAM99B was associated with hypoxia, we further explored the effects of FAM99A and FAM99B on the glucose metabolic reprogramming of HCC cells. Overexpression of FAM99A and FAM99B maintained the pH environment of HCCLM3 cells, and knockdown of FAM99A and FAM99B further reduced the pH of HCCLM3 cell supernatant (Figure 3A, *p* < 0.05). Notably, overexpression of FAM99A and FAM99B effectively reduced glucose levels in HCCLM3 cells and elevated them in conditioned medium, and knockdown of FAM99A and FAM99B exhibited opposite modulation patterns (Figure 3B,C, *p* < 0.05). Consistent with expectations, overexpression of FAM99A and FAM99B significantly decreased lactate and ATP contents in hypoxic HCCLM3 cells, while knockdown of FAM99A and FAM99B obtained the opposite results (Figure 3D,E, *p* < 0.05). Overexpressing FAM99A and FAM99B significantly diminished the expression of glycolysis‐related proteins (HK, PFKL and PKM2) in hypoxic HCCLM3 cells compared to controls, while knockdown of FAM99A and FAM99B favored their expression (Figure 3F, *p* < 0.05). Further, this study examined the effects of FAM99A and FAM99B on the metabolic pattern and rate of hepatocellular carcinoma cells using Seahorse assay. Overexpression of FAM99A and FAM99B observably reduced OCR, ECAR, and glycoPER in HCCLM3 cells, especially overexpression of FAM99B, whereas knockdown of FAM99A and FAM99B showed the opposite regulatory pattern in HEPG2 cells (Figure 4A,B). Statistical analysis revealed that overexpression of FAM99A and FAM99B effectively decreased basal glycoPER and basal PER and elevated mitoOCR/glycoPER in HCCLM3 cells, whereas knockdown of FAM99A and FAM99B led to opposite results in HEPG2 cells (Figure 4C,D). The above results demonstrated that FAM99A and FAM99B inhibited the glucose metabolic reprogramming in HCC cells and this process was prevented by the hypoxic environment.

### 3.3 Identification of FAM99A and FAM99B‐Associated DEGs and DE‐miRNAs

**FIGURE 5:**
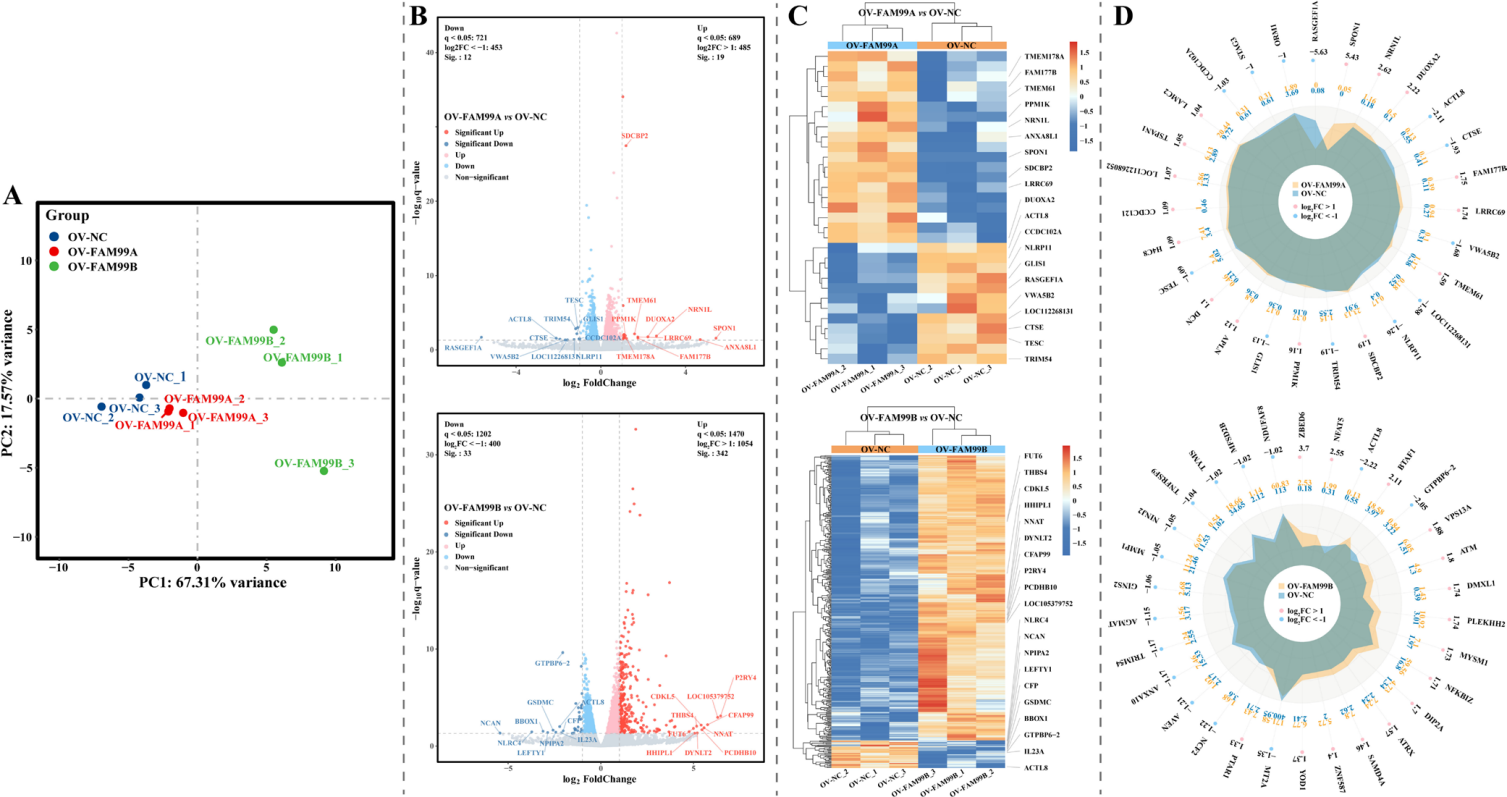
Identification of FAM99A and FAM99B‐related DEGs. (A) PCA analysis was performed to demonstrate the differences in the mRNA expression profiles of each group of samples. (B) Volcano plot displaying the DEGs of the OV‐FAM99A or OV‐FAM99B group compared to the OV‐NC group. The threshold is *p* < 0.05 for calibration and log_2_FC > 1 for absolute values. (C) Heat map showing the expression profiles of all DEGs and their clustering. (D) Radar plot illustrating the top 30 DEGs for log_2_FC with absolute values.

**FIGURE 6:**
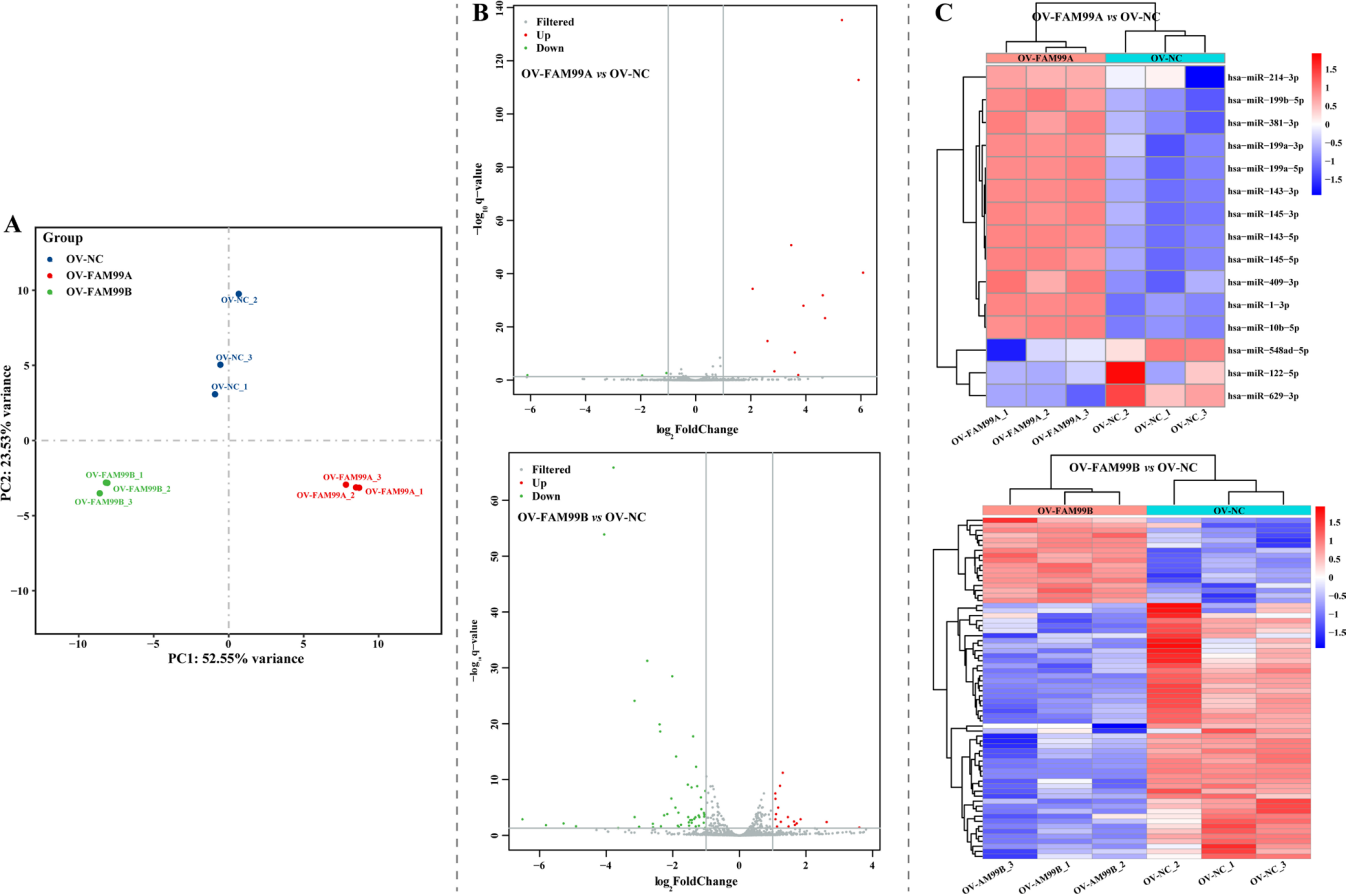
Identification of FAM99A‐ and FAM99B‐related DE‐miRNAs. (A) Differences in miRNA expression profiles among groups of samples were analyzed based on PCA. (B) Volcano plots are displayed for all DE‐miRNAs with thresholds of adjusted *p* < 0.05 and absolute values of log_2_FC > 1. (C) Hotmap reveals the clustering of all samples and DE‐miRNAs. The above graph shows the DE‐miRNAs in the OV‐FAM99A group compared to the OV‐NC group, and the following graph displays the DE‐miRNAs in the OV‐FAM99B group compared to the OV‐NC group.

To investigate the mechanisms by which FAM99A and FAM99B regulate proliferation, metastasis, and glucose metabolism reprogramming in HCC cells, we subjected HCCLM3 cells to transcriptome and smallRNA sequencing. PCA revealed that the expression profiles of mRNAs and miRNAs were closer together within groups and further apart between groups (Figures 5A and 6A). As shown in Figures 5B and 6A, compared to controls, a total of 31 DEGs (containing 12 significantly downregulated and 19 significantly upregulated DEGs) and 15 DE‐miRNAs (containing 3 significantly downregulated and 12 significantly upregulated DE‐miRNAs) were present in HCCLM3 cells overexpressing FAM99A, and a total of 375 DEGs (containing 33 significantly downregulated and 342 significantly upregulated DEGs) and 68 DE‐miRNAs (containing 51 significantly downregulated and 17 significantly upregulated DE‐miRNAs) were identified in HCCLM3 cells overexpressing FAM99B. The heat map demonstrates that the expression profiles of both DEGs and DE‐miRNAs of each sample have a good clustering (Figures 5C and 6C), which further validates the results of PCA. Radar plot displaying DEGs with log_2_FC TOP30 (Figure 5D). It is suggested that these DEGs and DE‐miRNAs may be involved in the regulation of HCC cell proliferation, metastasis, and glucose metabolism reprogramming with FAM99A and FAM99B.

### 3.4 Enrichment Analysis of FAM99A‐ and FAM99B‐Related DEGs and DE‐miRNA Targets

**FIGURE 7:**
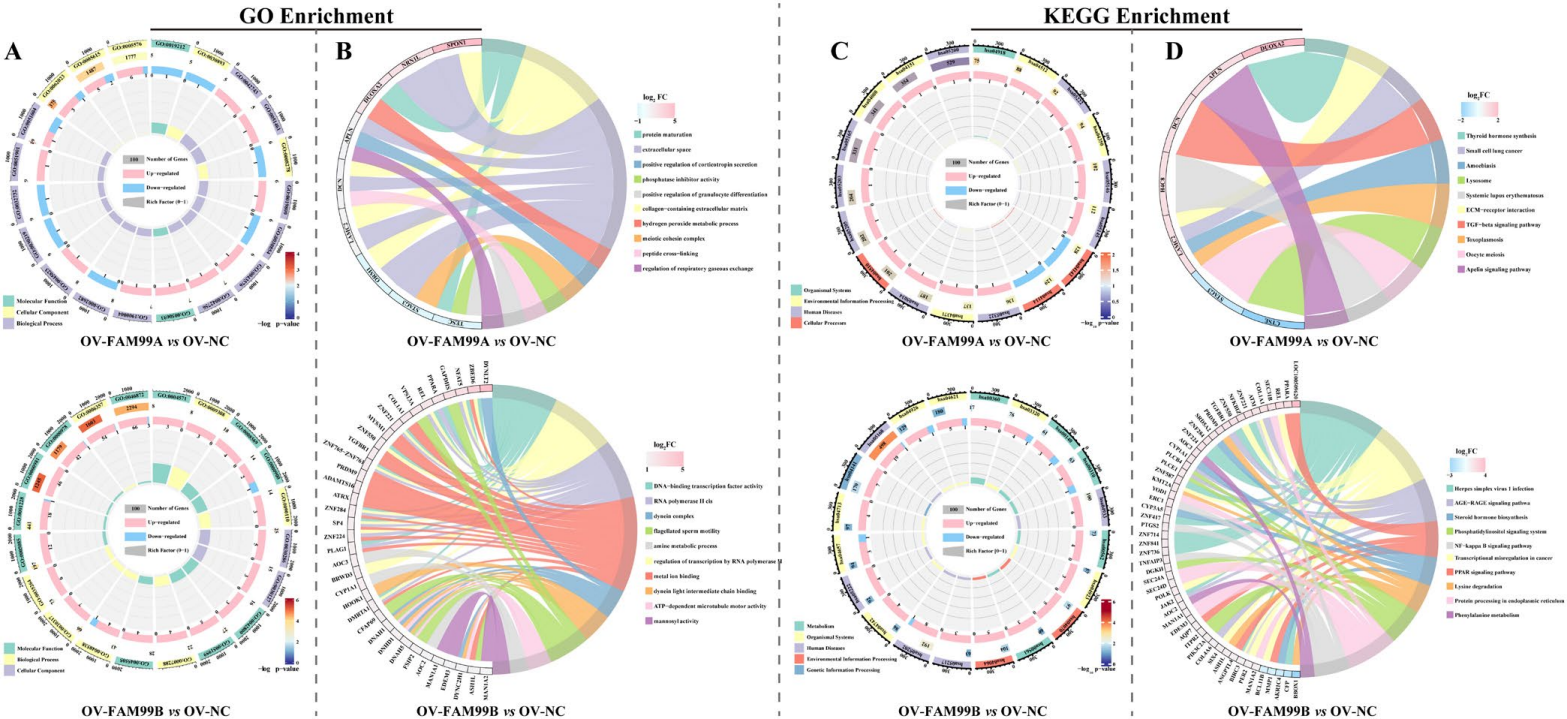
GO and KEGG enrichment analysis of FAM99A and FAM99B‐associated DEGs. (A, B) GO enrichment results of DEGs in the OV‐FAM99A or OV‐FAM99B groups compared to the OV‐NC group. (A) Circos plot showing the top 20 GO terms with −log_10_ *p* value. (B) Chord Diagram illustrating the 10 GO terms associated with the top log_2_FC with absolute value ranked DEGs. (C) Circos plot and (D) Chord Diagram exhibiting the results of KEGG pathway enrichment analysis for all DEGs.

Further, we analyzed the possible functions and pathways involved in the FAM99A and FAM99B‐associated DEGs and DE‐miRNA targets based on GO and KEGG. FAM99A and FAM99B‐related DEGs were enriched to 75 (“hydrogen peroxide metabolic process,” “regulation of immune system process,” “negative regulation of serine‐type endopeptidase activity,” etc.) and 147 (“extrinsic apoptotic signaling pathway,” “cellular response to ATP,” “response to hyperoxia,” etc.) GO terms (Figure 7A,B). FAM99A‐related DEGs mainly involved in KEGG pathway including “extracellular matrix (ECM)‐receptor interaction,” “TGF‐beta signaling pathway,” “PI3K‐Akt signaling pathway,” etc., and FAM99B‐related DEGs are mainly concerned with “phenylalanine metabolism,” “phosphatidylinositol signaling system,” “NF‐kappa B signaling pathway,” and “transcriptional misregulation in cancer” (Figure 7C,D).

**FIGURE 8:**
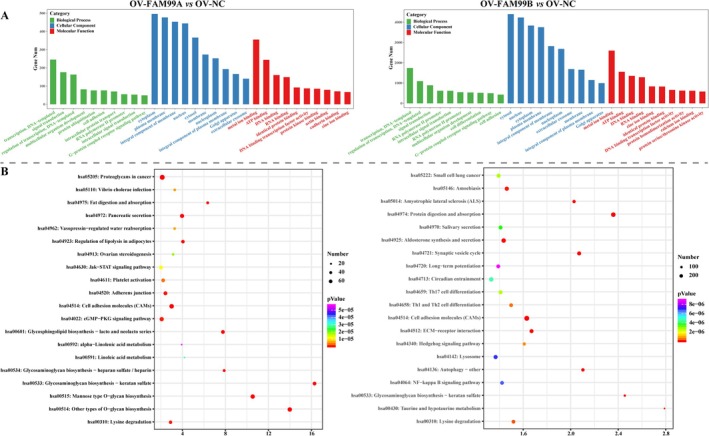
GO and KEGG enrichment analysis of FAM99A and FAM99B‐associated DE‐miRNAs. (A) Bar graphs illustrating the TOP10 BP, CC, and MF terms in GOs obtained from the enrichment of all DE‐miRNA targets. (B) Bubble plots showing the top 20 KEGG pathways with −log_10_ *p* value obtained by enrichment of DE‐miRNA targets. The left side shows the enrichment results of DE‐miRNA targets in the OV‐FAM99A group compared to the OV‐NC group, and the right side indicates the enrichment results of DE‐miRNA targets in the OV‐FAM99B group compared to the OV‐NC group.

Similarly, FAM99A‐associated DE‐miRNAs target enriched GO term and KEGG pathways including “N‐acetylglucosamine deacetylase activity,” “glucose transmembrane transport,” “ATPase activity,” “glycolysis/gluconeogenesis,” “citrate cycle,” “pentose and glucuronate interconversions,” etc. (Figure 8A,B). FAM99B‐related DE‐miRNAs targets are mainly involved in “execution phase of apoptosis,” “negative regulation of cell cycle arrest,” “pentose phosphate pathway,” “fructose and mannose metabolism,” “galactose metabolism,” etc. (Figure 8A,B).

This indicated that FAM99A‐ and FAM99B‐related DEGs and DE‐miRNAs may be participating in mediating processes such as cell cycle, apoptosis, metastasis, immunity, inflammation, ECM remodeling and metabolic reprogramming, which further confirmed the regulation of these phenotypes by FAM99A and FAM99B in HCC.

### 3.5 Construction of FAM99A‐ and FAM99B‐Associated PPI and ceRNA Networks

**FIGURE 9:**
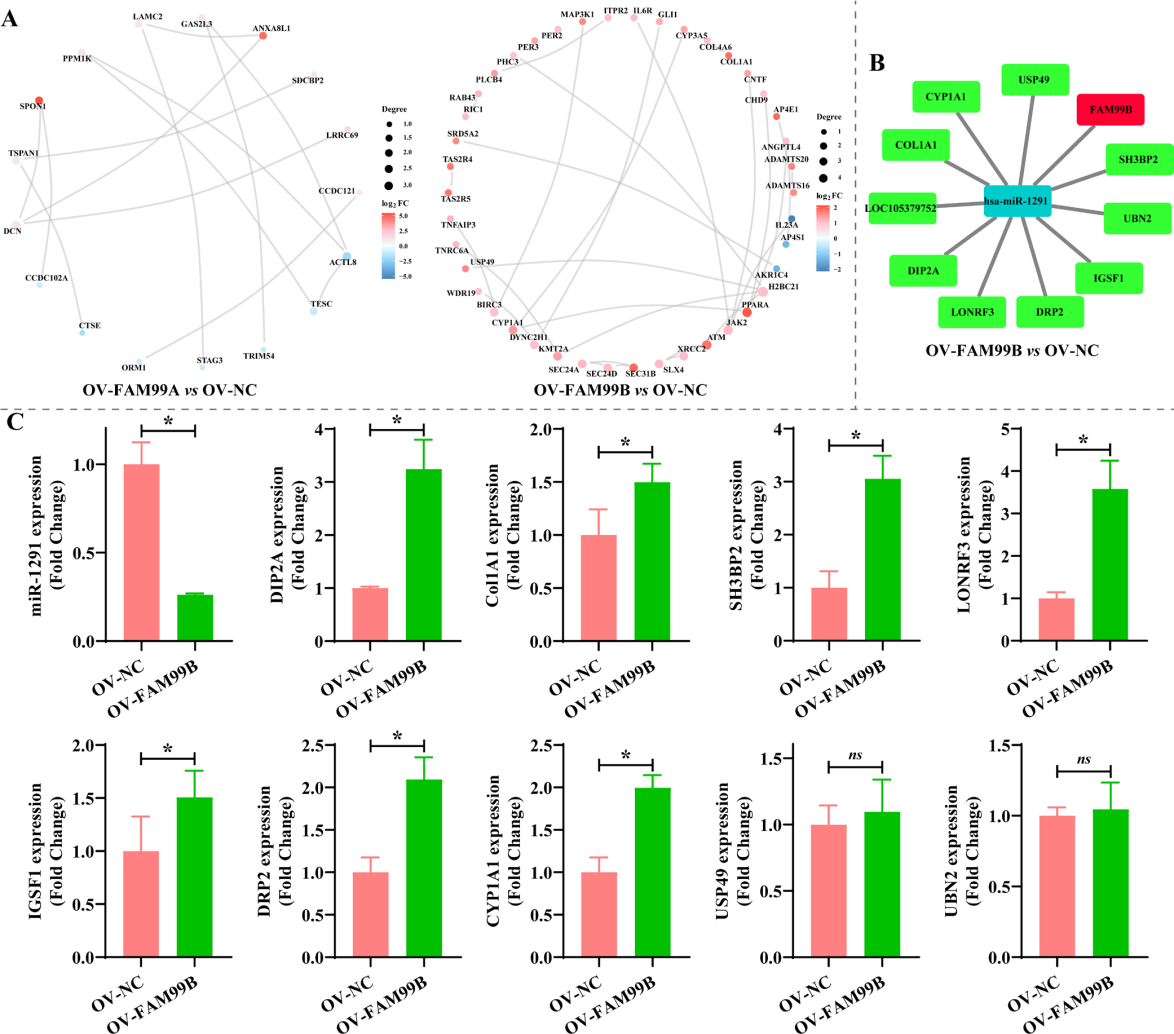
Construction of FAM99A and FAM99B‐related PPI and ceRNA networks. (A) PPI network of DEGs in the (Left) OV‐FAM99A or (right) OV‐FAM99B group compared to the OV‐NC group. (B) FAM99B‐related ceRNA network constructed based on DEGs and DE‐miRNAs from the OV‐FAM99B group compared to the OV‐NC group. (C) Differential expression of DEGs and DE‐miRNAs in the FAM99B‐associated ceRNA network was verified using RT‐qPCR. Data that conformed to normal distribution were statistically analyzed using Student's *t* test; otherwise, Mann–Whitney *U* test was used. *N* = 3, * represents *p* < 0.05 and ns means no significant difference.

To understand the possible regulatory mechanisms of FAM99A and FAM99B on these DEGs and DE‐miRNAs, we constructed PPI and ceRNA networks. The PPI networks constructed by FAM99A‐ and FAM99B‐associated DEGs contained 17 and 42 nodes, respectively, with *DCN*, *H2BC21*, *JAK2*, *SEC24D*, and *PPARA* being the genes with the most edges (Figure 9A). An effective FAM99A‐associated ceRNA network was not constructed due to the low number of FAM99A‐associated DEGs and DE‐miRNAs. As exhibited in Figure 9B, the FAM99B‐associated ceRNA network contained one DE‐miRNA and 10 DEGs. RT‐qPCR results demonstrated that the expression of DE‐miRNAs and DEGs in the FAM99B‐associated ceRNA network was consistent with the sequencing results, except for *USP48* and *UBN2* (Figure 9C and Table S3, *p* < 0.05). It is indicated that DEGs and DE‐miRNAs in PPI and ceRNA networks may be potential regulatory mechanisms for FAM99A and FAM99B‐mediated HCC phenotypes.

## 4 Discussion

Hepatocellular carcinoma (HCC) is a serious malignancy that usually occurs in the setting of chronic liver disease, such as cirrhosis and hepatitis B. As with many other cancers, glucose metabolic reprogramming is closely associated with the development and progression of HCC, and it provides for the proliferation and survival of HCC cells. We found that FAM99A and FAM99B expression was inhibited in hypoxic HCC cells and that this facilitated HCC cell proliferation, metastasis, and glucose metabolic reprogramming. Moreover, 31 DEGs and 15 DE‐miRNAs, 375 DEGs and 68 DE‐miRNAs were identified in HCC cells overexpressing FAM99A or FAM99B, respectively. Enrichment analysis demonstrated that these DEGs and DE‐miRNAs targets may mediate processes such as cell proliferation, apoptosis, metastasis, immunity, ECM remodeling, and metabolic reprogramming, which further confirmed the role of FAM99A or FAM99B in the HCC phenotype.

In fact, some roles of FAM99A and FAM99B as liver‐specific lncRNAs in HCC have been demonstrated. Bioinformatics analysis by Mo et al. [16, 17, 20] confirmed that FAM99A and FAM99B are significantly low expressed in HCC tissues and may be involved in metabolic pathways, and that patients with high expression of FAM99A and FAM99B are predicted to have a better prognosis. In addition, their functional assays also demonstrated that overexpression of FAM99A and FAM99B contributed to the inhibition of HCC cell proliferation, migration, and invasion [16, 17]. These results are similar to ours, but we further substantiated that FAM99A and FAM99B are tumor suppressors under hypoxic conditions, which further enriches the value of FAM99A and FAM99B as biomarkers in the treatment of HCC. HCC cells are generally under hypoxic conditions, which stimulate glucose metabolic reprogramming of tumor cells to maintain sufficient energy and vital activity required by tumor cells [29, 30]. Since hypoxia inhibited the expression of FAM99A and FAM99B in this study, we further explored the effect of FAM99A and FAM99B on the glucose metabolic reprogramming of HCC. Indeed, Zhao et al. [20] also revealed that hypoxia and CoCl2 significantly inhibited FAM99A expression in HCC cells and that downregulation of FAM99A promotes HCC cell metastasis via miRNA‐92a. This is consistent with our results, and we further supported that FAM99B has similar effects and that their downregulation also facilitates glucose metabolic reprogramming in HCC cells. Notably, Icarisitin, as a therapeutic agent for HCC, was found to promote the expression of FAM99A, thereby blocking the JAK1/STAT2 pathway that mediates HCC cell proliferation and GLUT3‐related glycolysis [31]. Undoubtedly, our findings provide a solid theoretical basis for the future application of FAM99A and FAM99B as therapeutic targets for HCC.

Noticeably, we identified hub gene such as *DCN*, *H2BC21*, *JAK2*, *SEC24D* and *PPARA*, and FAM99B‐associated ceRNA network consisting of miR‐1291, *CYP1A1*, *USP48*, etc., by transcriptome and smallRNA sequencing. Interestingly, the role of these genes or miR‐1291 in HCC has been partially confirmed. For example, miR‐1291 was demonstrated to positively regulate Glypican‐3, which is considered the most promising HCC serum indicator to play an oncogenic role in HCC [32]. Nevertheless, the opposite result was obtained by Marion et al. [33]. This is puzzling, although the present study found miR‐1291 to be significantly under‐expressed in HCC cells overexpressing FAM99B. The expression and mutation of JAK2 is associated with glucose metabolic reprogramming in myeloproliferative neoplasms [34], type 3 diabetes‐induced liver tumors [35] and mesenchymal stem cells [36], and the JAK2/Stat3 pathway facilitates the progression of HCC [37, 38]. DCN was identified as a tumor suppressor in HCC, and it regulates the proliferation and metastasis of HCC cells [39, 40, 41]. A bioinformatic analysis revealed that CYP1A1 is a marker associated with prognosis and metabolism in thyroid cancer [42]. PPARA has been demonstrated to be associated with glucose metabolic reprogramming in glioblastoma [43], bladder cancer [44] and lung cancer [45]. It is noteworthy that USP48 is upregulated by METTL14 with M6A modification, which facilitates the stabilization of SIRT6 expression to inhibit glycolysis in HCC [46]. It is suggested that these DEGs and DE‐miRNAs are most likely potential targets for FAM99A and FAM99B to mediate HCC phenotype.

In conclusion, this study demonstrated that FAM99A and FAM99B expression was inhibited in HCC cells under a hypoxic environment, which facilitated the growth, metastasis, and glucose metabolic reprogramming of HCC cells. Nevertheless, we lack in vivo experiments to demonstrate these effects of FAM99A and FAM99B on HCC, and a large number of potential mechanisms revealed by transcriptome and smallRNA sequencing remain to be further confirmed.

## Author Contributions

C.S., G.W., P.M., H.Z., L.L., X.M., X.L., and Y.Z. conceived and designed the experiments; C.S., G.W., P.M., H.Z., L.L., and X.M. performed the experiments; C.S. and G.W. analyzed and interpreted the data; P.M., H.Z., L.L., and X.M. contributed reagents/materials/analysis tools; C.S. wrote the original draft; G.W., P.M., H.Z., L.L., X.M., X.L., and Y.Z. reviewed and edited the draft. All authors have read and agreed to the published version of the manuscript.

## Ethics Statement

The authors have nothing to report.

## Consent

All authors have read and agreed to the published version of the manuscript.

## Conflicts of Interest

The authors declare no conflicts of interest.

## Supporting information


Table S1.

## Data Availability

The data are available on request from the corresponding author.

## References

[1] A. Forner , M. Reig , and J. Bruix , “Hepatocellular Carcinoma,” Lancet 391, no. 10127 (2018): 1301–1314. 29307467 10.1016/S0140-6736(18)30010-2

[2] C. Xia , X. Dong , H. Li , et al., “Cancer Statistics in China and United States, 2022: Profiles, Trends, and Determinants,” Chinese Medical Journal 135, no. 5 (2022): 584–590. 35143424 10.1097/CM9.0000000000002108 PMC8920425

[3] R. L. Siegel , K. D. Miller , N. S. Wagle , and A. Jemal , “Cancer Statistics, 2023,” CA: A Cancer Journal for Clinicians 73, no. 1 (2023): 17–48. 36633525 10.3322/caac.21763

[4] D. Anwanwan , S. K. Singh , S. Singh , V. Saikam , and R. Singh , “Challenges in Liver Cancer and Possible Treatment Approaches,” Biochimica Et Biophysica Acta. Reviews on Cancer 1873, no. 1 (2020): 188314. 31682895 10.1016/j.bbcan.2019.188314 PMC6981221

[5] T. Torimura and H. Iwamoto , “Treatment and the Prognosis of Hepatocellular Carcinoma in Asia,” Liver International 42, no. 9 (2022): 2042–2054. 34894051 10.1111/liv.15130

[6] D. Liu and T. Song , “Changes in and Challenges Regarding the Surgical Treatment of Hepatocellular Carcinoma in China,” Bioscience Trends 15, no. 3 (2021): 142–147. 33716267 10.5582/bst.2021.01083

[7] L. Chen , X. W. Chen , X. Huang , B. L. Song , Y. Wang , and Y. Wang , “Regulation of Glucose and Lipid Metabolism in Health and Disease,” Science China. Life Sciences 62, no. 11 (2019): 1420–1458. 31686320 10.1007/s11427-019-1563-3

[8] P. Vaupel , H. Schmidberger , and A. Mayer , “The Warburg Effect: Essential Part of Metabolic Reprogramming and Central Contributor to Cancer Progression,” International Journal of Radiation Biology 95, no. 7 (2019): 912–919. 30822194 10.1080/09553002.2019.1589653

[9] K. Hönigova , J. Navratil , B. Peltanova , H. H. Polanska , M. Raudenska , and M. Masarik , “Metabolic Tricks of Cancer Cells,” Biochimica Et Biophysica Acta. Reviews on Cancer 1877, no. 3 (2022): 188705. 35276232 10.1016/j.bbcan.2022.188705

[10] C. Schiliro and B. L. Firestein , “Mechanisms of Metabolic Reprogramming in Cancer Cells Supporting Enhanced Growth and Proliferation,” Cells 10, no. 5 (2021): 1056. 33946927 10.3390/cells10051056 PMC8146072

[11] I. Martínez‐Reyes and N. S. Chandel , “Cancer Metabolism: Looking Forward,” Nature Reviews. Cancer 21, no. 10 (2021): 669–680. 34272515 10.1038/s41568-021-00378-6

[12] G. J. Yoshida , “Metabolic Reprogramming: The Emerging Concept and Associated Therapeutic Strategies,” Journal of Experimental & Clinical Cancer Research 34 (2015): 111. 26445347 10.1186/s13046-015-0221-y PMC4595070

[13] L. Fagerberg , B. M. Hallström , P. Oksvold , et al., “Analysis of the Human Tissue‐Specific Expression by Genome‐Wide Integration of Transcriptomics and Antibody‐Based Proteomics,” Molecular & Cellular Proteomics 13, no. 2 (2014): 397–406. 24309898 10.1074/mcp.M113.035600 PMC3916642

[14] B. Xu , X. Geng , X. Liu , and Y. Liu , “Long Non‐Coding RNA FAM99A Modulated YAP1 to Affect Trophoblast Cell Behaviors in Preeclampsia by Sponging miR‐134‐5p,” Brazilian Journal of Medical and Biological Research 53, no. 12 (2020): e9732. 33111745 10.1590/1414-431X20209732 PMC7584153

[15] T. He , Y. Qiao , Y. Lv , J. Wang , R. Hu , and Y. Cao , “lncRNA FAM99A Is Downregulated in Preeclampsia and Exerts a Regulatory Effect on Trophoblast Cell Invasion, Migration and Apoptosis,” Molecular Medicine Reports 20, no. 2 (2019): 1451–1458. 31173227 10.3892/mmr.2019.10350

[16] M. Mo , S. Liu , X. Ma , et al., “A Liver‐Specific lncRNA, FAM99B, Suppresses Hepatocellular Carcinoma Progression Through Inhibition of Cell Proliferation, Migration, and Invasion,” Journal of Cancer Research and Clinical Oncology 145, no. 8 (2019): 2027–2038. 31243545 10.1007/s00432-019-02954-8 PMC11810180

[17] M. Mo , X. Ma , Y. Luo , et al., “Liver‐Specific lncRNA FAM99A May Be a Tumor Suppressor and Promising Prognostic Biomarker in Hepatocellular Carcinoma,” BMC Cancer 22, no. 1 (2022): 1098. 36289466 10.1186/s12885-022-10186-2 PMC9609286

[18] C. J. Petry , A. Koulman , L. Lu , et al., “Associations Between the Maternal Circulating Lipid Profile in Pregnancy and Fetal Imprinted Gene Alleles: A Cohort Study,” Reproductive Biology and Endocrinology 16, no. 1 (2018): 82. 30157874 10.1186/s12958-018-0399-x PMC6116391

[19] M. Sun , S. Lv , and J. Zhong , “In Silico Analysis of the Association Between Long Non‐Coding RNA Family With Sequence Similarity 99 Member A (FAM99A) and Hepatic Cancer,” IET Systems Biology 17 (2023): 83–94. 36854891 10.1049/syb2.12062 PMC10116027

[20] B. Zhao , K. Ke , Y. Wang , et al., “HIF‐1α and HDAC1 Mediated Regulation of FAM99A‐miR92a Signaling Contributes to Hypoxia Induced HCC Metastasis,” Signal Transduction and Targeted Therapy 5, no. 1 (2020): 118. 32636357 10.1038/s41392-020-00223-6 PMC7341733

[21] C. A. Schneider , W. S. Rasband , and K. W. Eliceiri , “NIH Image to ImageJ: 25 Years of Image Analysis,” Nature Methods 9, no. 7 (2012): 671–675. 22930834 10.1038/nmeth.2089 PMC5554542

[22] K. J. Livak and T. D. Schmittgen , “Analysis of Relative Gene Expression Data Using Real‐Time Quantitative PCR and the 2(‐Delta Delta C(T)) Method,” Methods 25, no. 4 (2001): 402–408. 11846609 10.1006/meth.2001.1262

[23] M. I. Love , W. Huber , and S. Anders , “Moderated Estimation of Fold Change and Dispersion for RNA‐Seq Data With DESeq2,” Genome Biology 15, no. 12 (2014): 550. 25516281 10.1186/s13059-014-0550-8 PMC4302049

[24] D. Betel , M. Wilson , A. Gabow , D. S. Marks , and C. Sander , “The MicroRNA.org Resource: Targets and Expression,” Nucleic Acids Research 36, no. Database Issue (2008): D149–D153. 18158296 10.1093/nar/gkm995 PMC2238905

[25] H. Mi , D. Ebert , A. Muruganujan , et al., “PANTHER Version 16: A Revised Family Classification, Tree‐Based Classification Tool, Enhancer Regions and Extensive API,” Nucleic Acids Research 49, no. D1 (2021): D394–D403. 33290554 10.1093/nar/gkaa1106 PMC7778891

[26] M. Kanehisa , Y. Sato , M. Kawashima , M. Furumichi , and M. Tanabe , “KEGG as a Reference Resource for Gene and Protein Annotation,” Nucleic Acids Research 44, no. D1 (2016): D457–D462. 26476454 10.1093/nar/gkv1070 PMC4702792

[27] D. Szklarczyk , A. L. Gable , K. C. Nastou , et al., “The STRING Database in 2021: Customizable Protein‐Protein Networks, and Functional Characterization of User‐Uploaded Gene/Measurement Sets,” Nucleic Acids Research 49, no. D1 (2021): D605–D612. 33237311 10.1093/nar/gkaa1074 PMC7779004

[28] P. Shannon , A. Markiel , O. Ozier , et al., “Cytoscape: A Software Environment for Integrated Models of Biomolecular Interaction Networks,” Genome Research 13, no. 11 (2003): 2498–2504. 14597658 10.1101/gr.1239303 PMC403769

[29] M. H. Bao and C. C. Wong , “Hypoxia, Metabolic Reprogramming, and Drug Resistance in Liver Cancer,” Cells 10, no. 7 (2021): 1715. 34359884 10.3390/cells10071715 PMC8304710

[30] D. Du , C. Liu , M. Qin , et al., “Metabolic Dysregulation and Emerging Therapeutical Targets for Hepatocellular Carcinoma,” Acta Pharmaceutica Sinica B 12, no. 2 (2022): 558–580. 35256934 10.1016/j.apsb.2021.09.019 PMC8897153

[31] X. Zheng , Y. Gou , Z. Jiang , A. Yang , Z. Yang , and S. Qin , “Icaritin‐Induced FAM99A Affects GLUT1‐Mediated Glycolysis via Regulating the JAK2/STAT3 Pathway in Hepatocellular Carcinoma,” Frontiers in Oncology 11 (2021): 740557. 34765550 10.3389/fonc.2021.740557 PMC8576446

[32] N. A. Hagag , Y. B. M. Ali , A. A. Elsharawy , and R. M. Talaat , “Clinical Impact of Circulated miR‐1291 in Plasma of Patients With Liver Cirrhosis (LC) and Hepatocellular Carcinoma (HCC): Implication on Glypican‐3 Expression,” Journal of Gastrointestinal Cancer 51, no. 1 (2020): 234–241. 31028536 10.1007/s12029-019-00234-9

[33] M. Maurel , S. Jalvy , Y. Ladeiro , et al., “A Functional Screening Identifies Five microRNAs Controlling Glypican‐3: Role of miR‐1271 Down‐Regulation in Hepatocellular Carcinoma,” Hepatology 57, no. 1 (2013): 195–204. 22865282 10.1002/hep.25994

[34] T. N. Rao , N. Hansen , J. Hilfiker , et al., “JAK2‐Mutant Hematopoietic Cells Display Metabolic Alterations That Can Be Targeted to Treat Myeloproliferative Neoplasms,” Blood 134, no. 21 (2019): 1832–1846. 31511238 10.1182/blood.2019000162 PMC6872961

[35] H. Jiang , Q. Yao , Y. An , L. Fan , J. Wang , and H. Li , “Baicalin Suppresses the Progression of Type 2 Diabetes‐Induced Liver Tumor Through Regulating METTL3/m(6)A/HKDC1 Axis and Downstream p‐JAK2/STAT1/Clevaged Capase3 Pathway,” Phytomedicine 94 (2022): 153823. 34763315 10.1016/j.phymed.2021.153823

[36] M. Yao , Z. Chen , X. He , et al., “Cross Talk Between Glucose Metabolism and Immunosuppression in IFN‐γ‐Primed Mesenchymal Stem Cells,” Life Science Alliance 5, no. 12 (2022): e202201493. 36260750 10.26508/lsa.202201493 PMC9463493

[37] T. Yang , R. Xu , J. Huo , et al., “WWOX Activation by Toosendanin Suppresses Hepatocellular Carcinoma Metastasis Through JAK2/Stat3 and Wnt/β‐Catenin Signaling,” Cancer Letters 513 (2021): 50–62. 34015398 10.1016/j.canlet.2021.05.010

[38] Y. Xiao , Y. Li , D. Shi , et al., “MEX3C‐Mediated Decay of SOCS3 mRNA Promotes JAK2/STAT3 Signaling to Facilitate Metastasis in Hepatocellular Carcinoma,” Cancer Research 82, no. 22 (2022): 4191–4205. 36112698 10.1158/0008-5472.CAN-22-1203

[39] C. H. Liu , B. R. Wu , Y. J. Ho , et al., “CHPF Regulates the Aggressive Phenotypes of Hepatocellular Carcinoma Cells via the Modulation of the Decorin and TGF‐β Pathways,” Cancers (Basel) 13, no. 6 (2021): 1261. 33809195 10.3390/cancers13061261 PMC8002199

[40] A. Reszegi , Z. Horváth , H. Fehér , et al., “Protective Role of Decorin in Primary Hepatocellular Carcinoma,” Frontiers in Oncology 10 (2020): 645. 32477937 10.3389/fonc.2020.00645 PMC7235294

[41] Y. Li , L. Gan , M. Lu , et al., “HBx Downregulated Decorin and Decorin‐Derived Peptides Inhibit the Proliferation and Tumorigenicity of Hepatocellular Carcinoma Cells,” FASEB Journal 37, no. 4 (2023): e22871. 36929160 10.1096/fj.202200999RR

[42] Q. Du , R. Zhou , H. Wang , et al., “A Metabolism‐Related Gene Signature for Predicting the Prognosis in Thyroid Carcinoma,” Frontiers in Genetics 13 (2022): 972950. 36685893 10.3389/fgene.2022.972950 PMC9846547

[43] T. T. T. Nguyen , E. Shang , C. Shu , et al., “Aurora Kinase A Inhibition Reverses the Warburg Effect and Elicits Unique Metabolic Vulnerabilities in Glioblastoma,” Nature Communications 12, no. 1 (2021): 5203. 10.1038/s41467-021-25501-x PMC8410792 34471141

[44] S. Logotheti , S. Marquardt , S. K. Gupta , et al., “LncRNA‐SLC16A1‐AS1 Induces Metabolic Reprogramming During Bladder Cancer Progression AS Target and Co‐Activator of E2F1,” Theranostics 10, no. 21 (2020): 9620–9643. 32863950 10.7150/thno.44176 PMC7449907

[45] G. Ye , H. Gao , X. Zhang , et al., “Aryl Hydrocarbon Receptor Mediates Benzo[a]Pyrene‐Induced Metabolic Reprogramming in Human Lung Epithelial BEAS‐2B Cells,” Science of the Total Environment 756 (2021): 144130. 33288249 10.1016/j.scitotenv.2020.144130

[46] L. Du , Y. Li , M. Kang , et al., “USP48 Is Upregulated by Mettl14 to Attenuate Hepatocellular Carcinoma via Regulating SIRT6 Stabilization,” Cancer Research 81, no. 14 (2021): 3822–3834. 33903120 10.1158/0008-5472.CAN-20-4163

