# Supplementary material for: Hypoxia‐Induced Suppression of FAM99A and FAM99B Contributes to the Development and Glucose Metabolic Reprogramming of Hepatocellular Carcinoma
Source: FASEB J. 2025 Jul 22;39(14):e70869. doi: 10.1096/fj.202501058R (PMC12282501; doi:10.1096/fj.202501058R)
Supplement: Supplementary file 1 — Table S1. [file FSB2-39-e70869-s001.docx]

**Supplementary table 1 shRNA sequences of FAM99A and FAM99B and lentiviral titers**

| NO. | Target sequence | Lentivirus titer (TU/mL) |
| --- | --- | --- |
| Sh-NC | TTCTCCGAACGTGTCACGT |  |
| Sh-FAM99A #1 | GCCGGGCCCGGCTCTGGTACT | 5×10^8^ |
| Sh-FAM99A #2 | GAAGGTGTGAATCAGTTTACA | 5×10^8^ |
| Sh-FAM99A #3 | GATGCGTGTGTGGGCTCATCC | 5×10^8^ |
| PGMLV-CMV-H_FAM99A |  | 1.14×10^8^ |
| Sh-FAM99B #1 | GCCGAGACATTTCGTAATTAG | 2.04×10^8^ |
| Sh-FAM99B #2 | GCCCACGACATCAGGTAAACA | 1.15×10^8^ |
| Sh-FAM99B #3 | GGCTAAGCTGGGAGCTTATCT | 1.17×10^8^ |
| PGMLV-CMV-H_FAM99B |  | 1.11×10^8^ |

**Supplementary table 2 Primer sequences of target genes**

| Target gene | Forward | Reverse |
| --- | --- | --- |
| GAPDH | GCTTGTGACCAGTGTAAC | GAGGTAGTCTGTAGATCATTAAC |
| U6 | AACGCTTCACGAATTTGCGT | CTCGCTTCGGCAGCACA |
| USP49 | CTATTCTTCGCTACCTACC | CTCCTTGACCATCTTCTG |
| CYP1A1 | GTGCTAAAGGTGCCAATG | GTCCTTGAACACCAACAG |
| COL1A1 | GTTGTGAAGTTAGAAGGTA | ATTCCAAGCAATCCATAG |
| DIP2A | CCGACATTGAATACATTGAA | TGATGCTTGCTGATAGAC |
| LONRF3 | CACATCCAGGCTATTAAG | AATTCTCCATTGACAAGT |
| DRP2 | ATCATTCTTCCTCAACAA | GGCTTATCTACAACCATA |
| IGSF1 | TTATGGAGACAGAAGTGA | CAGAAGTAAGATGATTATGAAC |
| UBN2 | GAACTCCTCTACCAAGTC | AATGCGATAGTTCCTCAC |
| SH3BP2 | GCTTGTGACCAGTGTAAC | GAGGTAGTCTGTAGATCATTAAC |
| FAM99A | TGGGAGCTTATCTTCCCCGA | CGCATCACAAAACAGCCACA |
| FAM99B | GGCCCACGACATCAGGTAAA | GGGGAAGATAAGCTCCCAGC |
| miR-1291 | ATTCCAGTGGCCCTGACTGA | GTCCTTTAGGCCTCTGCTTGA |

**Supplementary table 3 Sequencing results of DEGs and DE-miRNAs**

| Gene ID | Log_2_FC | Adj. *P* value | Regulation |
| --- | --- | --- | --- |
| miR-1291 | -1.468342987 | 0.001708827 | Down |
| DIP2A | 1.704857801 | 2.76E-14 | Up |
| COL1A1 | 1.840650366 | 0.002547468 | Up |
| SH3BP2 | 1.266617456 | 2.52E-13 | Up |
| LONRF3 | 1.271980272 | 8.51E-07 | Up |
| IGSF1 | 1.244904858 | 0.001085758 | Up |
| DRP2 | 1.074625599 | 0.033249835 | Up |
| CYP1A1 | 1.432489057 | 0.000207575 | Up |
| USP49 | 1.633322238 | 8.91E-11 | Up |
| UBN2 | 1.867587828 | 6.99E-08 | Up |
